# Overexpression of the *Zygophyllum xanthoxylum* Aquaporin, *ZxPIP1;3*, Promotes Plant Growth and Stress Tolerance

**DOI:** 10.3390/ijms22042112

**Published:** 2021-02-20

**Authors:** Mengzhan Li, Mingfa Li, Dingding Li, Suo-Min Wang, Hongju Yin

**Affiliations:** State Key Laboratory of Grassland Agro-Ecosystems, Key Laboratory of Grassland Livestock Industry Innovation, Ministry of Agriculture and Rural Affairs, College of Pastoral Agriculture Science and Technology, Lanzhou University, Lanzhou 730020, China; limzh2015@lzu.edu.cn (M.L.); limf19@lzu.edu.cn (M.L.); lidd2017@lzu.edu.cn (D.L.); smwang@lzu.edu.cn (S.-M.W.)

**Keywords:** aquaporin, *Zygophyllum xanthoxylum*, plant growth, abiotic stress

## Abstract

Drought and salinity can result in cell dehydration and water unbalance in plants, which seriously diminish plant growth and development. Cellular water homeostasis maintained by aquaporin is one of the important strategies for plants to cope with these two stresses. In this study, a stress-induced aquaporin, ZxPIP1;3, belonging to the PIP1 subgroup, was identified from the succulent xerophyte *Zygophyllum xanthoxylum*. The subcellular localization showed that ZxPIP1;3-GFP was located in the plasma membrane. The overexpression of *ZxPIP1;3* in Arabidopsis prompted plant growth under favorable condition. In addition, it also conferred salt and drought tolerance with better water status as well as less ion toxicity and membrane injury, which led to more efficient photosynthesis and improved growth vigor via inducing stress-related responsive genes. This study reveals the molecular mechanisms of xerophytes’ stress tolerance and provides a valuable candidate that could be used in genetic engineering to improve crop growth and stress tolerance.

## 1. Introduction

Abiotic stress factors, such as drought and high salinity, are recognized as major environmental threats that may break plant water balance and result in tissue dehydration, thus negatively impacting plant growth and development. As sessile organisms, plants have gradually evolved various strategies to control water flux to cope with environmental constraints [1,2].

Aquaporins (AQPs), a type of major intrinsic protein (MIP) spreading across the plant kingdom, play important roles in maintaining cellular water homeostasis [3,4]. AQPs contain six membrane-spanning domains and two highly conserved Asn-Pro-Ala (NPA) motifs [5]. According to their amino acid sequences and subcellular localization, AQPs can be classified into five subfamilies, including plasma membrane intrinsic proteins (PIPs), tonoplast intrinsic proteins (TIPs), nodulin-26 intrinsic proteins (NIPs), small basic intrinsic proteins (SIPs), and X-intrinsic proteins (XIPs) [6]. PIPs, located at the plasma membrane, are the largest subfamily of plant AQPs and play key roles in transcellular water transport. This subfamily can be subdivided into two groups, PIP1s and PIP2s [7], which are different in the length of N- and C- termini and in water conductivity. PIP2s, commonly possessing a longer C-termini and a shorter N-termini, are more efficient in water movement. However, PIP1s, with shorter C-termini and longer N-termini, have less water conductivity but possess the ability to transport various uncharged small molecule [8,9]. Numerous studies have showed that PIPs are involved in response to salt and drought stresses, and the ectopical expression of some *PIP*s confers abiotic stress tolerance to plants [8]. The overexpression of *OsPIP1-1* and *OsPIP2-2*, two salt and drought-inducible *PIP*s, resulted in a higher salt and drought tolerance of Arabidopsis [10]. In addition, the ectopical expression of *MdPIP1;3* increased the fruit size of tomato and enhanced the drought tolerance of transgenic plants [11]. The overexpression of a salt-inducible *PIP*, *TaAQP8*, increased the salt stress tolerance of transgenic tobacco with promoted root growth [12]. However, a majority of current studies about AQPs mainly focus on glycophytes and rarely concentrate on xerophytes or halophytes containing specific traits generated during their long-term evolution in extremely severe environments [13,14].

*Zygophyllum xanthoxylum*, a kind of succulent xerophyte belonging to Zygophyllaceae, is widely spread in arid and semiarid land in northwestern China [15]. For its remarkable vitality to survive under adverse drought condition, *Z. xanthoxylum* is often used in sand-fixing as well as water and soil conservation in the desert [16]. Previous studies showed that this species can absorb Na^+^ from low salt soil and compartmentalize them into vacuoles as a low-cost osmoregulation substance, which helps *Z. xanthoxylum* maintain lower osmotic potential to absorb water under drought condition. All these studies focused on ion transporters participating in the salt and drought stress tolerance of *Z. xanthoxylum*, while the functions of AQPs in the course of water transportation and cellular water homeostasis maintenance remains unknown [15,17,18,19]. Previously, in order to understand the mechanisms of *Z. xanthoxylum* to cope with severe environment, 50 mM NaCl-treated and −0.5 MPa-treated transcriptome datasets of *Z. xanthoxylum* roots were analyzed [20,21]. An *AQP*, *ZxPIP1;3*, whose expression was induced under salt and osmotic treatment, was screened.

In this study, *ZxPIP1;3* was cloned, and its expression pattern under salt and osmotic treatment was identified by qRT-PCR. In addition, the transient expression of ZxPIP1;3-GFP fusion protein was used to investigate the subcellular localization in *Nicotiana benthamiana*. Furthermore, ectopical overexpression transgenic Arabidopsis was generated to evaluate the roles of ZxPIP1;3 in plant growth and stress tolerance.

## 2. Results

### 2.1. ZxPIP1;3 Is Induced under Osmotic and Salt Treatment

**Figure 1:**
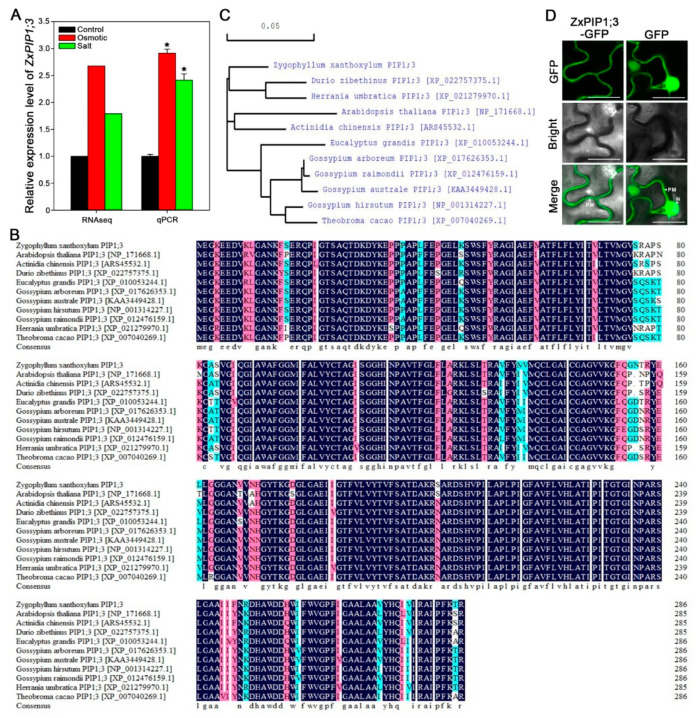
*ZxPIP1;3* encodes a PIP1 protein whose expression level is related with osmotic and salt treatments. (**A**) qRT-PCR validation of RNA sequencing data in *Z. xanthoxylum* roots under osmotic stress or salt treatment for 6 h. (**B**,**C**), alignment (**B**) and phylogenetic analysis (**C**) of ZxPIP1;3 with other known PIP1 proteins. Dark blue, pink and aqua indicate that the homology levels of these amino acids are 100%, more than 75% and more than 50% respectively. (**D**) Subcellular localization of ZxPIP1;3-GFP in epidermal cells of tobacco leaves. GFP driven by *CaMV35S* promoter served as control. PM, plasma membrane. N, nucleus. Green fluorescence represents GFP. Bar = 10 μm. For (**A**), asterisks indicate significant differences from control condition. Data shown are means of three independent biological replicates (* *p* < 0.05, one way ANOVA).

To verify the transcriptome data, 3-week-old *Z. xanthoxylum* seedlings were treated with −0.5 MPa osmotic stress or 50 mM NaCl treatment (Figure 1A). The expression level of *ZxPIP1;3* was significantly increased after 6-h treatment, which confirmed that *ZxPIP1;3* was stress-inducible and suggested that ZxPIP1;3 may participate in plants’ salt and drought stress response.

### 2.2. ZxPIP1;3 Encodes an AQP of PIP1 Subgroup

The full length of the *ZxPIP1;3* open reading frame was 864 bp, encoding 287 amino acids residues (Figure S1A). ZxPIP1;3 contained six putative transmembrane α-helices (Figure S1B). The results of multiple sequence alignment and phylogenetic tree analysis using full-length amino acid sequence indicated that ZxPIP1;3 was highly homologous to DzPIP1;3 (*Durio zibethinus*) and HuPIP1;3 (*Herrania umbratica*) (Figure 1B,C). Through subcellular localization using transient expression driven by the *CaMV35S* promoter in *N. benthamiana* leaves, ZxPIP1;3-GFP was detected at the plasma membrane (Figure 1D).

### 2.3. Overexpression of ZxPIP1;3 Promotes Plant Growth

To study the performance of ZxPIP1;3 in plant growth and abiotic stress tolerance, *35S::ZxPIP1;3-FLAG* was transformed into Arabidopsis. The expression levels of *ZxPIP1;3* in transgenic lines was detected, and two lines, *OE2* and *OE3*, with different expression level were selected for further analysis (Figure S2).

**Figure 2:**
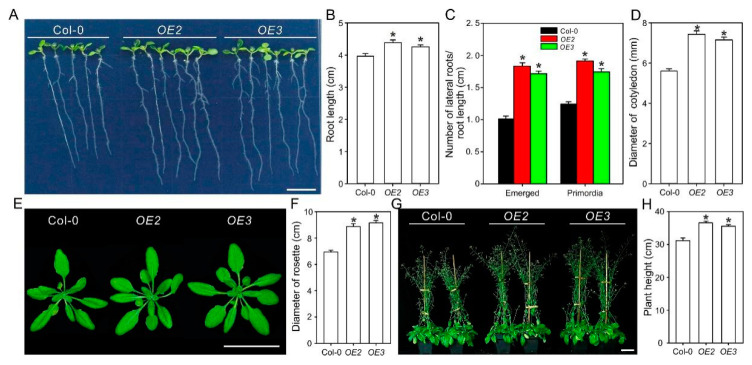
ZxPIP1;3 plays positive roles in plant growth. (**A**) Phenotypes of wild-type (Col-0) and *ZxPIP1;3* overexpression lines (*OE2* and *OE3*) grow vertically on 1/2 Murashige and Skoog (MS) medium for 7 days after germination. Bar = 1 cm. (**B**–**D**) Primary root length (**B**), number of emerged lateral roots and lateral root primordia (**C**) as well as cotyledon diameter (**D**) of plants treated as described in the legend of (**A**). (**E**) Phenotypes of 4-week-old seedlings grown on soil culture. Bar = 5 cm. (**F**) Rosette leaves diameter of plants treated as described in the legend of (**E**). (**G**) Phenotypes of 7-week-old seedlings on soil culture. Bar = 5 cm. (**H**) Height of plants treated as described in the legend of (**G**). For (**B**–**D**), (**F**,**H**), asterisks indicate significant differences from Col-0 (*n* = 13 per column. * *p* < 0.05, one way ANOVA).

Col-0 and *OE2*, *OE3* grown vertically on 1/2 Murashige and Skoog (MS) solid medium for 7 days after germination were used to evaluate the effect of ZxPIP1;3 on root growth and development. Comparing with wild type, primary roots of transgenic lines were longer with more emerged lateral root and lateral root primordia (Figure 2A–C), which indicated that *ZxPIP1;3* overexpression promoted root growth under favorable conditions. We further tested the roles of ZxPIP1;3 in shoot growth. The cotyledon of transgenic lines was larger than those of wild-type plants (Figure 2A,D). For 4-week-old seedlings, *OE2* and *OE3* grow better than Col-0 with larger rosette leaves (Figure 2E,F). Additionally, for plants during the reproductive stage, transgenic lines were taller compared with Col-0 (Figure 2G,H). Thus, it was evident that *ZxPIP1;3* overexpression could significantly promote plant growth.

### 2.4. ZxPIP1;3 Overexpression Improves Salt Tolerance of Transgenic Arabidopsis

To investigate the role of ZxPIP1;3 in response to salt stress, wild-type and transgenic Arabidopsis were grown on 1/2 MS with 150 mM NaCl. Under salt stress treatment, primary roots of *OE2* and *OE3* were longer than those of wild-type plants (Figure S3A). This result indicated that *ZxPIP1;3* transgenic seedlings were less sensitive to salt stress.

**Figure 3:**
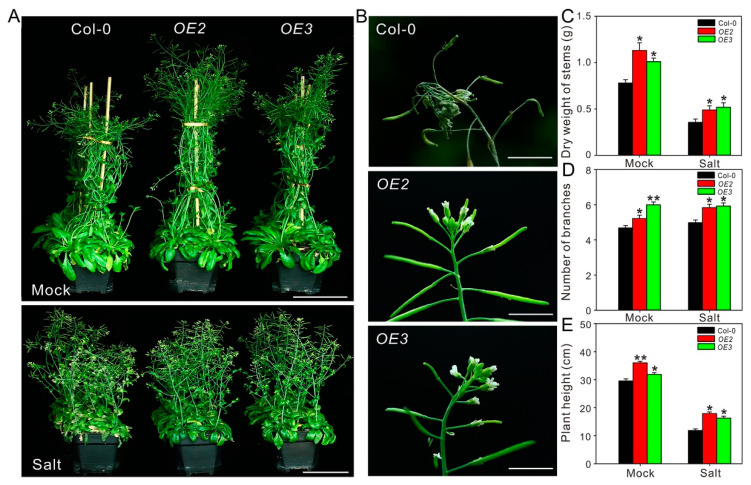
Overexpression of *ZxPIP1;3* improves growth vigor under salt treatment. (**A**) Phenotypes of soil-cultured seedlings treated with (Salt) or without (Mock) 100 mM NaCl for 20 days. Bar = 10 cm. (**B**) Phenotypes of shoot apices of plants under salt treatment as described in (**A**). Bar = 1 cm. (**C**–**E**) Dry weight of stems (**C**), number of branches (**D**) and plant height (**E**) of plants described in the legend of (**A**). For (**C–E**), asterisks indicate significant differences from Col-0 (*n* = 13 per column. * *p* < 0.05, ** *p* < 0.01, one way ANOVA).

**Figure 4:**
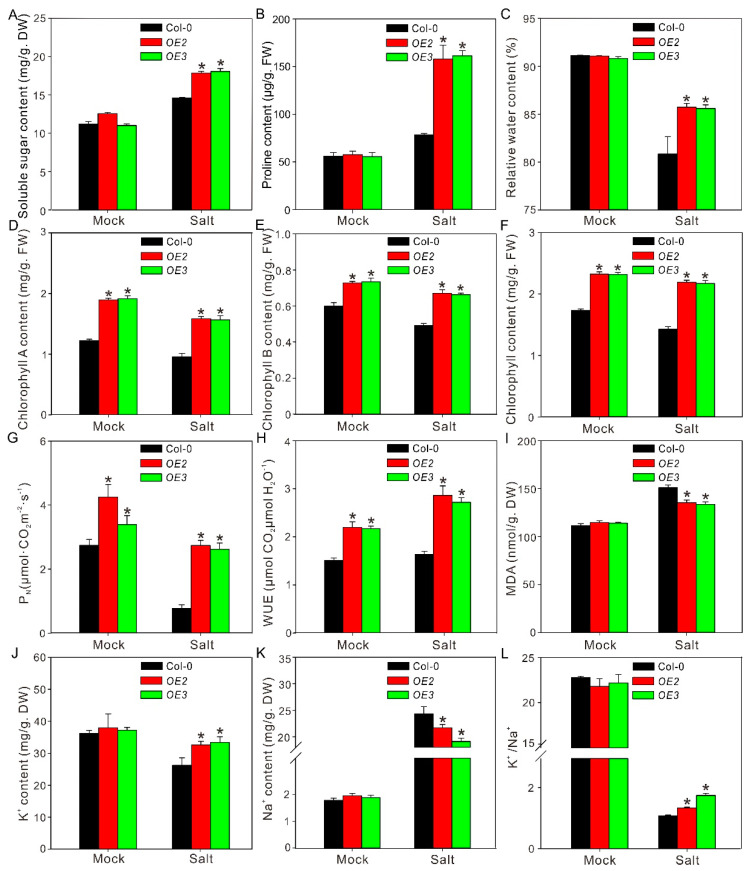
Several physiological parameters of the salt-treated wild-type and transgenic plants. Soluble sugar content (**A**), proline content (**B**), relative water content (**C**), chlorophyll A content (**D**), chlorophyll B content (**E**), chlorophyll content (**F**), net photosynthetic rate (Pn) (**G**), water-use efficiency (WUE) (**H**), content of malondialdehyde (MDA) (**I**), K^+^ content (**J**), Na^+^ content (**K**), and K^+^/Na^+^ (**L**) were tested. Asterisks indicate significant differences from Col-0 (Values are mean ± SE of three replicates. * *p* < 0.05, one way ANOVA).

To further confirm the function of ZxPIP1;3 in salt stress tolerance, 4-week-old plants were irrigated with 100 mM NaCl for 20 days. Under favorable condition, transgenic plants grow better (Figure 3A). Under salt stress condition, the wild-type plants were etiolated and aborted with sere leaves and inflorescence, while the leaves of transgenic plants were still green, and the shoot apices of them grow well without abortion (Figure 3A,B). In addition, the stems of *OE2* and *OE3* were longer and heavier with more branches than wild type (Figure 3C–E). Physiological parameters including the content of organic osmoregulation substance, relative water content, content of chlorophyll, net photosynthetic rate (Pn), water-use efficiency (WUE) and malondialdehyde (MDA) content as well as K^+^/Na^+^ ratio were also measured (Figure 4). Salinity has osmotic effects on plants, which lead to water deficiency [22]. To overcome physiological drought, a plant may synthesize organic osmotic substances, such as soluble sugar and proline, to increase water potential. Under salt treatment, transgenic plants contained more soluble sugar and proline than wild type, which resulted in much higher relative water content (Figure 4A–C). The stability of chlorophyll is sensitive to plant water status, and higher relative water content can protect chlorophyll from degradation [2]. Comparing with Col-0, the chlorophyll content of *OE2* and *OE3* was much higher under both normal condition and salt stress treatment, which was consistent with the phenotype (Figure 3A and Figure 4D–F). Consequently, osmotic homeostasis and higher chlorophyll content can also contribute to higher Pn and WUE in *OE2* and *OE3* than Col-0 after salt treatment (Figure 4G,H). As a final product of cell membrane lipid peroxidation, MDA is a good indicator of oxidative damage [23]. MDA content was significantly lower in *OE2* and *OE3* than Col-0 (Figure 4I), which implicated that membrane damage in transgenic lines was not as severe as those in wild type. Salinity can also cause ion toxicity on plants. Moreover, Na^+^ at high concentration competes for sites of transporters, which is necessary for K^+^ uptake [22]. There was no difference of K^+^ and Na^+^ content between transgenic and wild-type lines under normal condition. However, after salt treatment, *OE2* and *OE3* contained more K^+^ and less Na^+^ than Col-0, which resulted in higher K^+^/Na^+^ ratio (Figure 4J–L). All these results indicated that *ZxPIP1;3* overexpression improved the salt tolerance of transgenic lines.

### 2.5. ZxPIP1;3 Overexpression Confers Drought Tolerance of Transgenic Plants

To verify the functions of ZxPIP1;3 in drought stress response, drought stress treatment was simulated by the cultivation of Col-0 and *OE2*, *OE3* on 1/2 MS solid medium containing 300 mM mannitol. Under osmotic stress treatment, primary roots of *OE2* and *OE3* were longer than those of Col-0 (Figure S3A). This result suggested that *ZxPIP1;3* overexpression decreased plants’ sensitivity to simulant drought stress.

**Figure 5:**
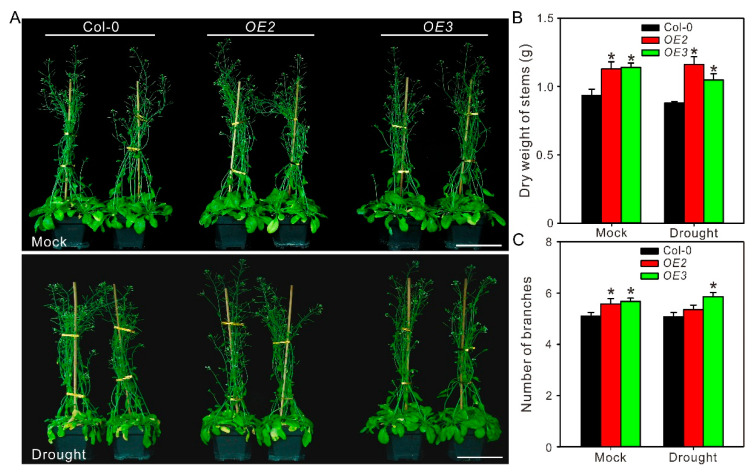
Overexpression of *ZxPIP1;3* improves growth vigor under drought treatment. (**A**) Phenotypes of soil-cultured seedlings treated with (Drought) or without (Mock) period dehydration. Bar = 10 cm. (**B**,**C**), Dry weight of stems (**B**) and number of branches (**C**) of plants treated as described in the legend of (**A**). For (**B**,**C**), asterisks indicate significant differences from Col-0 (*n* = 13 per column. * *p* < 0.05, one way ANOVA).

**Figure 6:**
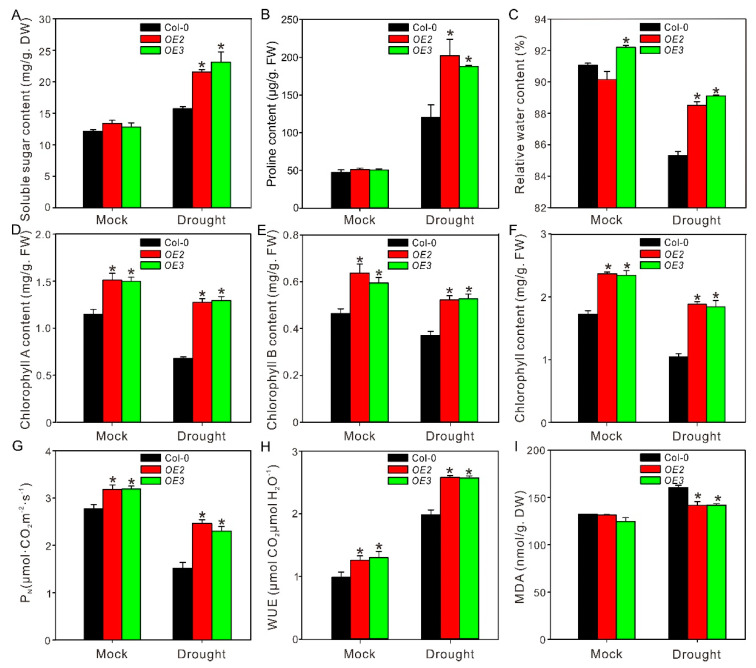
Several physiological parameters of the drought-treated wild-type and transgenic plants. Soluble sugar content (**A**), proline content (**B**), relative water content (**C**), chlorophyll A content (**D**), chlorophyll B content (**E**), chlorophyll content (**F**), Pn (**G**), WUE (**H**), and content of MDA (**I**) were tested. Asterisks indicate significant differences from Col-0 (Values are mean ± SE of three replicates. * *p* < 0.05, one way ANOVA).

Further, the drought tolerance of wild-type and transgenic lines were evaluated in soil culture. Col-0 and *ZxPIP1;3* overexpression plants were grown under well-watered condition for 4 weeks before subjected to drought treatment. After 7 days of water withdrawal, Col-0 began to wilt, while *OE2* and *OE3* grow well with unfolded leaves (Figure S3B). To further evaluate the effect of drought stress on different lines, plants treated with dehydration were watered normally for 7 days for recovery. After that, these plants were subjected to 7 days drought treatment again. Plants were photographed (Figure 5A) and then harvested for analyzing physiological parameters [19]. After period drought treatment, the leaves of Col-0 were etiolated and wilted, whereas *ZxPIP1;3* transgenic lines did not wilt as severely as Col-0 (Figure 5A). In addition, stems’ dry weight and the branch numbers of transgenic plants were higher than those of wild type (Figure 5B,C), which indicated that *OE2* and *OE3* showed a stronger growth vigor in comparison to Col-0 under water shortage. Physiological parameters including organic osmoregulation substance content, relative water content, chlorophyll content, and the Pn and WUE as well as MDA content of wild-type and transgenic lines were also measured (Figure 6). After period drought treatment, the contents of organic osmoregulation substance, including soluble sugar and proline, as well as relative water content in transgenic plants were higher than those of wild type (Figure 6A–C). Photosynthesis is influenced by water status and chlorophyll content. In addition, the accumulation of chlorophyll is also related with water status. Compared with wild type, a higher relative water content prevented the chlorophyll of transgenic lines from degradation, which resulted in higher chlorophyll concentration, Pn, and WUE (Figure 6D–H). Water deficiency can also lead to cell membrane destabilization. Under drought treatment, the MDA content of *OE2* and *OE3* was less than those of Col-0 (Figure 6I). All these results indicated that *ZxPIP1;3* overexpression conferred drought tolerance in transgenic plants.

### 2.6. Expression Level of Stress-Related Genes Is Increased in ZxPIP1;3 Transgenic Plants under Stress Treatment Compared with Wild-Type Plants

**Figure 7:**
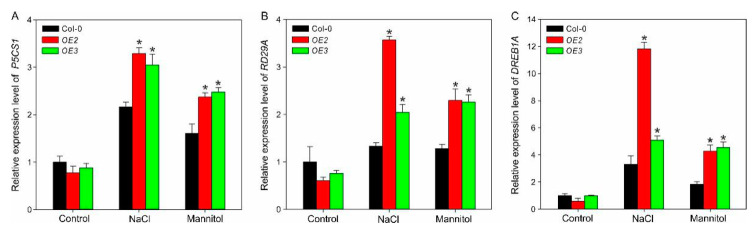
Relative expression levels of stress-related genes in wild-type and transgenic plants. The expression levels of *P5CS1* (**A**), *RD29A* (**B**), and *DREB1A* (**C**) were tested in 7-day-old wild-type (Col-0) and transgenic plants (*OE2*, *OE3*) under normal (Control), salt (NaCl), and osmotic (Mannitol) treatments. Asterisks indicate significant differences from Col-0. Data shown are means of three independent biological replicates. * *p* < 0.05, one way ANOVA.

To assess the implication of ZxPIP1;3 in the abiotic stress response pathway, the expression of three genes participating in stress response were analyzed via qRT-PCR (Figure 7). Δ1-pyrroline-5-carboxylate synthetase 1 (P5CS1) plays vital roles in proline biosynthesis [24]. The expression level of *P5CS1* in all lines increased under salt and osmotic stresses, and it was higher in transgenic lines compared with wild-type plants (Figure 7A). The expression level of *Response-to-Dehydration 29A* (*RD29A*), an ABA-induced gene related with responsiveness to drought, salt, and cold [25], was significantly higher in transgenic plants comparing with Col-0 under osmotic and salt stress (Figure 7B). *DEHYDRATION-RESPONSIVE ELEMENT-BINDING PROTEIN 1A* (*DREB1A*) is an APETALA2/ethylene-responsive element-binding factor (AP2/ERF)-type transcription factor involved in plant abiotic stress response [26]. The expression of *DREB1A* was remarkably enhanced in *OE2* and *OE3* under the stress-treated condition (Figure 7C).

## 3. Discussion

*Z. xanthoxylum* is widely distributed throughout the desert region of northwestern China, where the mean annual precipitation is usually less than 200 mm [27]. Previous studies have demonstrated that the main strategy for *Z. xanthoxylum* to cope with the extremely arid environment is to absorb Na^+^ from the low salt soil and compartmentalize them into vacuoles. Na^+^ can be used as a low-cost osmoregulator to decrease the osmotic potential, which helps *Z. xanthoxylum* absorb water under drought stress [17,18,27]. Most studies concerning the stress tolerance of this species were focused on the process of sodium uptake and accumulation [17,18,27]. Various ion transporters and channels were cloned and characterized [15,19]. However, mechanisms of the water influx that result from Na^+^-accumulation are obscure. As a majority of transmembrane water flux is dependent on AQPs, the first AQP from *Z. xanthoxylum*, ZxPIP1;3, was isolated, and its functions in plant growth as well as abiotic stress tolerance were evaluated in the present study.

To cope with stressful conditions and the growing demand of food, it is vital to develop cultivars with higher yields and improved tolerance to abiotic stress via genetic engineering breeding [28,29]. However, most identified genes play opposing roles in stress tolerance and plant growth, such as *C repeat/dehydration-responsive element binding factor 1* (*CBF1*) and *DWARF AND DELAYED FLOWERING 1* (*DDF1*), whose overexpression conferred stress tolerance at the expense of growth [30,31,32]. However, in this study, the overexpression of *ZxPIP1;3* can not only promote growth under normal condition (Figure 2) but also decrease the inhibition of salt and drought stress on it (Figure 3 and Figure 5), demonstrating that ZxPIP1;3 plays positive roles in plant growth as well as stress tolerance. Previous studies indicated that AQPs exert an effect on plant growth via impacting water absorption. In addition, the uptake of some nutrients can also be accompanied by water flux through AQPs [33]. Thus, ZxPIP1;3 is an optimal candidate for crop breeding.

Both salinity and water shortage trigger cell dehydration. It is important for plants to retain water from the environment under salt and drought stress. We observed that the relative water content of *ZxPIP1;3* transgenic plants was higher than those of wild type under stress treatment (Figure 4C and Figure 6C), indicating the enhanced ability of transgenic plants to retain water. Similar phenotypes were also reported via studying other *AQP*s transgenic plants, such as Arabidopsis overexpressing *PIP1;1* from banana and potato overexpressing *StPIP1* [34,35]. To investigate the mechanism involved in this process, the content of organic osmotic substances was measured, which were synthesized to adjust osmotic potential. The soluble sugar content of transgenic plants was higher than that of wild type (Figure 4A and Figure 6A), implying that *ZxPIP1;3* overexpression increased the ability of osmotic regulation. This result was consistent with the overexpression of *HvPIP2;5* [36] and *TsPIP1;3* [37], which also play positive roles in osmotic regulation. Plants also synthesize proline to adjust osmotic potential. P5CS participates in proline biosynthesis via reducing glutamate to glutamate semialdehyde [24]. Studies showed that the overexpression of *ZmPIP1;1* and *ScPIP1* can increase the accumulation of proline in transgenic plants via inducing the expression of *P5CS*, which led to enhanced stress tolerance [36,38,39]. In this study, consistent with previous studies, the expression level of *P5CS1* and the accumulation of proline in transgenic plants were also higher than those in wild-type plants (Figure 4B, Figure 6B and Figure 7A). Abiotic stresses induce a rapid accumulation of ROS, which leads to the cell membrane damage [23]. To estimate the membrane injury level, the content of MDA, a product of lipid oxidation, was measured. It was observed that the MDA content of transgenic plants was less than that of wild type (Figure 4I and Figure 6I), indicating that the membrane damage suffered by *ZxPIP1;3* overexpressing plants under dehydration was not as severe as wild type. Our results were consistent with previous studies demonstrating that PIPs participated in reducing membrane damage under different stress [37,40,41]. The water balance mediated by PIPs results in a relatively stable physiological status, which may lead to reduced protein and lipid peroxidation followed by decreased MDA content and membrane damage. High salinity decreases the growth rate of plants via increasing cellular Na^+^ concentration. To avert the toxic effects of sodium in cytosol, plants intend to compartmentalize Na^+^ into vacuoles. The transport of Na^+^ into vacuoles is regulated by Na^+^/H^+^ antiporters and vacuolar H^+^-translocating enzymes, whose activation is related to the stability of the membrane [42]. We surmised that the reduced membrane damage contributed by *ZxPIP1;3* overexpression may help to maintain the functions of transporters localized in the cell membrane and promote the vacuolar Na^+^ compartmentation, which reduced the cytotoxic effects of sodium.

Previous studies showed that most *AQP*s transgenic plants with enhanced stress tolerance exhibit a higher expression level of stress-responsive genes under stress-treated condition comparing with wild-type lines [34,38,43,44], which is consistence with our results (Figure 7). Salt and drought stresses are water-related, which can change osmotic gradients. Even though the certain mechanisms remained unclear, studies suggested that AQPs may act as detectors of osmotic gradients and relay information to signaling chains through protein conformation or interaction with downstream signaling elements [45,46,47]. Thus, there was no significant difference in the expression level of stress-responsive genes between three lines under optimal condition (Figure 7). However, after salt or osmotic treatment, the overexpression of *ZxPIP1;3* may enhance plants’ response to these stress signals and result in the higher expression level of stress-related genes indirectly (Figure 7), which elevated plants’ stress tolerance.

In conclusion, we identified a stress-induced *AQP*, *ZxPIP1;3*, from *Z. xanthoxylum* and demonstrated that ZxPIP1;3 not only improved growth vigor under favorable condition but also conferred salt and drought tolerance via enhancing the capacity of water retention as well as diminishing membrane injury and ion toxicity. This study reveals the molecular mechanisms of xerophytes’ stress tolerance and provides a theoretical basis for environmental protection in the desert area as well as discovers a valuable candidate for crop breeding.

## 4. Materials and Methods

### 4.1. Plant Materials and Growth Conditions

*Z. xanthoxylum* seeds were germinated on wet filter paper at 25 °C in the dark. After germination, seedlings were transformed into a hole plate containing quartz sand, whose grain size was about 0.5–0.8 cm, and them irrigated with modified Hoagland solution as Ma et al. described [21] every 3 days. Seedlings were grown in greenhouse at 28 °C/23 °C (day/night) under a 16-h-light/8-h-dark cycle with the flux density 800 μmol m^−2^ s^−1^. The relative humidity was about 65–70%. Arabidopsis used in this study was in ecotype Columbia-0 (Col-0) background. Arabidopsis seeds were vernalized in sterile water at 4 °C for 3 days before being grown on turfy soil in a greenhouse with relative humidity 65–75% at 22 °C under a 16-h-light/8-h-dark cycle with the flux density of 100–120 μmol m^−2^ s^−1^.

### 4.2. Expression Pattern Analysis

Three-week-old *Z. xanthoxylum* plants were used for different treatment for 6 h as follows. (i) Control: seedlings were irrigated with modified Hoagland solution; (ii) Salt treatment: seedlings were irrigated with modified Hoagland solution containing 50 mM NaCl; (iii) Osmotic stress: seedlings were irrigated with modified Hoagland solutions supplemented with sorbitol to adjust osmotic potential to -0.5 MPa. Roots of seedlings in each condition were collected and frozen by liquid nitrogen immediately.

Total RNA was extracted by using an RNAprep Pure Plant Plus Kit (Polysaccharides & Polyphenolics-rich) (TIANGEN, Beijing, China), and cDNA was synthesized from DNase-pretreated RNA using a PrimeScript™ RT reagent Kit with gDNA Eraser (TaKaRa Biotechnology, Beijing, China). qRT-PCR was performed in triplicate on three bio-replicates with a StepOne Real-Time PCR Thermocylcer (Applied Biosystems, Foster City, CA, USA) using the Power SYBR™ Green Master Mix (TaKaRa Biotechnology, Beijing, China). *ZxACTIN* (GenBank accession no. EU019550) was used as the internal control gene [15,19,20,21]. Sequences of primers are listed in Table S1. The 2^−ΔΔCt^ method was used to determine the relative expression level [48].

### 4.3. Cloning of ZxPIP1;3 and Sequence Analysis

Total RNA was extracted from roots of 3-week-old *Z. xanthoxylum* subjected to −0.5 MPa osmotic stress. The cDNA sequence of *ZxPIP1;3* (GenBank accession no. MW590708) was amplified by using a SMART RACE cDNA Amplification Kit (TaKaRa Biotechnology, China). Sequences of primers are listed in Table S1.

Nucleotide and amino acid sequences were analyzed by using DNAMAN (DNAMAN Inc., San Ramon, CA, USA). Transmembrane helices were predicted by TMHMM Server v 2.0 (http://www.cbs.dtu.dk/services/TMHMM/ (accessed on 1 January 2021)). Full-length amino acid sequences of PIP1;3 from Arabidopsis, *Actinidia chinensis*, *D. zibethinus*, *Eucalyptus grandis*, *Gossypium arboretum*, *Gossypium austral*, *Gossypium hirsutum*, *Gossypium raimondii*, *H. umbratical*, and *Theobroma cacao* were obtained from the NCBI database (https://www.ncbi.nlm.nih.gov/ (accessed on 1 January 2021)). DNAMAN were used for sequence alignment and polygenetic analysis.

### 4.4. ZxPIP1;3 Expression Vector Construction

*ZxPIP1;3* coding sequence was amplified with primers listed in Table S1. The product was cloned into a pDONR-ZERO vector by BP reaction and then inserted into the binary vectors, *pBIB-BASTA-35S-GWR-GFP* and *pBIB-BASTA-35S-GWR-FLAG*, by LR reaction. These binary constructs were introduced into the *Agrobacterium tumefaciens* strain GV3101 separately.

### 4.5. Subcellular Localization

GV3101 harboring *pBIB-BASTA-35S-ZxPIP1;3-GFP* was used for subcellular localization. GV3101 harboring *pCAMBIA1302* was used as control. After incubating in Luria–Bertani broth containing 10 mM MES (pH 5.7) and 20 mM acetosyringone at 28 °C overnight with shaking, cells were collected and adjusted to an OD600 of 0.6 with MS liquid media containing 10 mM MES (pH 5.7), 10 mM MgCl_2_, and 150 mM acetosyringone. After incubating at 28 °C for 2 h, the resuspension solution was injected into *N. benthamiana*. Leaves were used for subcellular localization analysis after 48-h infiltration.

### 4.6. Transgenic Plants Generation

The transformation of Arabidopsis Col-0 plants was performed via the floral dipping method by using GV3101 harboring *pBIB-BASTA-35S-GWR-FLAG*. The expression level of *ZxPIP1;3-FLAG* in transgenic plants with BASTA resistance was investigated via semi-quantitative RT-PCR. In addition, the expression levels of *ZxPIP1;3-FLAG* in wild type and *OE2*, *OE3* were also evaluated via qRT-PCR. To avoid nonspecific amplification, the reverse primers for semi-quantitative RT-PCR and qRT-PCR were designed by using the *FLAG* sequence. *AtACTIN 2* (AT3G18780) was used as the internal control for both semi-quantitative RT-PCR and qRT-PCR. Primers are listed in Table S1. The third generation of homozygous plants were used for further analysis.

### 4.7. Root and Shoot Growth Analysis

For lateral root growth analysis, seeds of wild-type and transgenic lines were plated on 1/2 MS solid medium and grown for 7 days after germination. Photographs were taken with a digital camera. The number of lateral root and lateral root primordia of 15 seedlings for each line were counted via an Olympus light microscope (magnification 100×), and 13 seedlings for each line were used for statistical analysis.

For primary root growth analysis, seeds of wild-type and transgenic plants were plated on 1/2 MS solid medium without or with 150 mM NaCl or 300 mM mannitol and grown for 7 days after germination. Photographs were taken with a digital camera. A primary root length of 15 seedlings for each line under respective treatment was measured by using Digimizer (MedCalc Software Ltd. Ostend, Belgium), and 13 seedlings for each line under respective treatment were used for statistical analysis.

For shoot growth analysis, cotyledon diameter, rosette leaves diameter and plant height, dry weight of stems, and number of branches were measured. For each parameter, 13 out of 15 individuals were used for statistical analysis for different lines.

### 4.8. Salt Tolerance Analysis of Transgenic Plants

For salt treatment with soil-grown plants, 4-week-old seedlings were irrigated with or without 100 mM NaCl for 20 days every 4 days. After being photographed with a digital camera, plants were harvested for physiological parameters analysis. The content of soluble sugar and proline, relative water content, chlorophyll content, and net photosynthetic rate, K^+^/Na^+^ ratio as well as malondialdehyde content were determined as described in previous studies, respectively [17,18,36,49,50].

### 4.9. Drought Tolerance Analysis of Transgenic Plants

For drought tolerance analysis in soil culture, 4-week-old plants were subjected with dehydration for 7 days and photographed with a digital camera (Figure S3B). To further evaluate the effect of drought stress on growth, drought-treated plants were irrigated normally for 7 days for recovery and treated with another 7 days’ water withdraw. Plants were photographed via a digital camera (Figure 5A) and then harvested for analyzing physiological parameters. The content of soluble sugar and proline, relative water content, chlorophyll content, and net photosynthetic rate, as well as malondialdehyde content were determined as previous studies, respectively [17,18,36,49,50].

### 4.10. Expression Pattern Analysis of Stress-Related Genes

Wild-type and transgenic lines grown on 1/2 MS solid medium for 7 days were treated as follows for 6 h: (i) Control: 1/2 MS liquid medium; (ii) Salt stress: 1/2 MS liquid medium containing 150 mM NaCl; (iii) Osmotic stress: 1/2 MS liquid medium containing 300 mM Mannitol. Roots of each treatment were collected, and total RNA were extracted. The transcript level of three stress-related genes *P5CS1* (AT2G39800), *RD29A* (At5G52310), and *DREB1A* (At4G25480) were evaluated, and *AtACTIN 2* (AT3G18780) was used as the internal control. The primer sequences used in this part are listed in Table S1.

### 4.11. Statistical Analysis

All analyses were performed by using IBM SPSS Statistics, version 22. (SPSS Inc., Chicago, IL, USA). All data shown as mean ± standard error of means were analyzed using one-way ANOVA, followed by Duncan’s test. Statistically significant mean values were denoted as * (*p* < 0.05) or ** (*p* < 0.01).

## Data Availability

Not applicable.

## Supplementary Materials

The following are available online at https://www.mdpi.com/1422-0067/22/4/2112/s1.
