# Supplementary material for: Overexpression of the Zygophyllum xanthoxylum Aquaporin, ZxPIP1;3, Promotes Plant Growth and Stress Tolerance
Source: Int J Mol Sci. 2021 Feb 20;22(4):2112. doi: 10.3390/ijms22042112 (PMC7924366; doi:10.3390/ijms22042112)
Supplement: Supplementary file 1 [file ijms-22-02112-s001.pdf]

Supplementary Materials

A

```
1    ATGGAAGGAAAAGAAGAGGATGTGAAGTTAGGAGCAAACAAGTTTTCAGAGAGGCAGCCAATAGGGACATCGGCACAGACAGACAAAGAC
1     M  E  G  K  E  E  D  V  K  L  G  A  N  K  F  S  E  R  Q  P  I  G  T  S  A  Q  T  D  K  D
91   TACAAGGAGCCACCACCAGCACCGTTGTTCGAGCCTGGAGAGCTAAAGTCATGGTCTTTCTACAGAGCTGGTATTGCTGAGTTCATGGCT
31    Y  K  E  P  P  P  A  P  L  F  E  P  G  E  L  K  S  W  S  F  Y  R  A  G  I  A  E  F  M  A
181  ACTTTCTTGTTCCTCTACATCACTGTCTTGACTGTCATGGGCGTAAGCCGGGCTCCTTCCAAGTGTGCCTCTGTTGGCATTCAAGGGATT
61    T  F  L  F  L  Y  I  T  V  L  T  V  M  G  V  S  R  A  P  S  K  C  A  S  V  G  I  Q  G  I
271  GCTTGGGCCTTTGGTGGCATGATCTTTGCTCTCGTCTACTGTACTGCCGGCATCTCAGGTGGACATATTAACCCAGCAGTGACATTTGGG
91    A  W  A  F  G  G  M  I  F  A  L  V  Y  C  T  A  G  I  S  G  G  H  I  N  P  A  V  T  F  G
361  TTGTTCTTGGCGAGGAAGTTGTCGCTTACAAGAGCGGTGTTTTACATGGTGATGCAGTGTCTTGGTGCTATTTGTGGTGCTGGAGTTGTC
121   L  F  L  A  R  K  L  S  L  T  R  A  V  F  Y  M  V  M  Q  C  L  G  A  I  C  G  A  G  V  V
451  AAGGGTTTCCAAGGGAATACAAGGTATGAGCTGCTTGGTGGTGGTGCTAATGTTGTCAACCATGGCTACACTAAGGGTGATGGTCTTGGT
151   K  G  F  Q  G  N  T  R  Y  E  L  L  G  G  G  A  N  V  V  N  H  G  Y  T  K  G  D  G  L  G
541  GCTGAGATTATTGGAACTTTTGTGCTTGTCTACACTGTGTTCTCTGCCACTGATGCCAAGAGAAGTGCCAGAGACTCTCACGTCCCTATT
181   A  E  I  I  G  T  F  V  L  V  Y  T  V  F  S  A  T  D  A  K  R  S  A  R  D  S  H  V  P  I
631  TTGGCTCCTCTGCCTATTGGATTTGCAGTGTTTTTGGTTCACTTGGCCACCATCCCAATCACAGGAACGGGCATCAACCCTGCTAGGAGT
211   L  A  P  L  P  I  G  F  A  V  F  L  V  H  L  A  T  I  P  I  T  G  T  G  I  N  P  A  R  S
721  CTCGGAGCTGCCATCATCTTCAACAGAGACCATGCATGGGATGACCACTGGATCTTCTGGGTTGGTCCCTTCATTGGAGCTGCTCTTGCT
241   L  G  A  A  I  I  F  N  R  D  H  A  W  D  D  H  W  I  F  W  V  G  P  F  I  G  A  A  L  A
811  GCTGTTTACCACCAGATTGTCATCAGAGCCATTCCATTCAAGACCAGGGCATAA
271   A  V  Y  H  Q  I  V  I  R  A  I  P  F  K  T  R  A  *
```

B

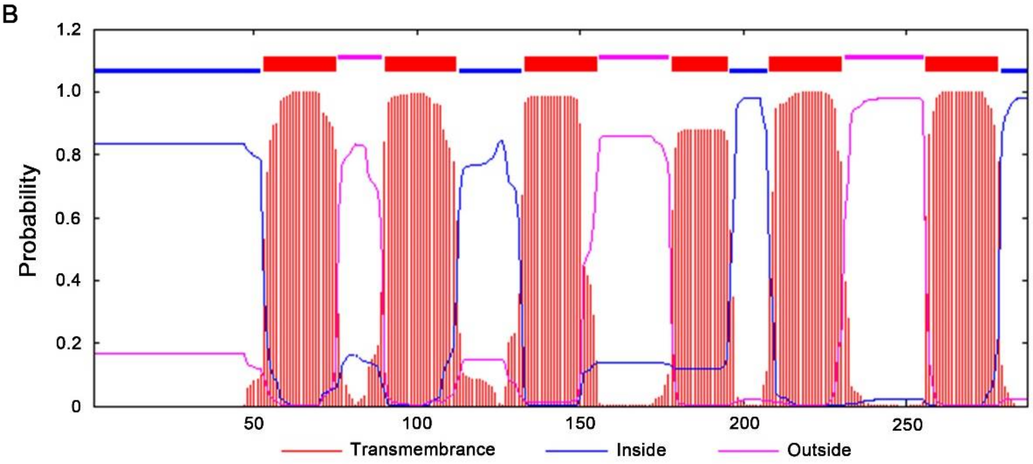

Figure S1. Coding sequence and transmembrane helices predication of *ZxPIP1;3*. **(A)** Full length of *ZxPIP1;3* coding sequence is 864 bp, encoding 287 amino acids. **(B)** ZxPIP1;3 protein contains 6 putative transmembrane domains.

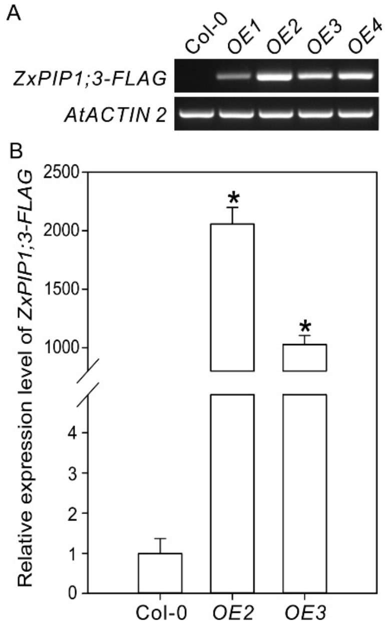

Figure S2. Generation of transgenic Arabidopsis plants. **(A)** Expression levels of *ZxPIP1;3-FLAG* in wild-type (Col-0) and transgenic plants (*OE1-4*) were evaluated via semi-quantitative RT-PCR.

(B) Relative expression levels of *ZxPIP1;3-FLAG* in Col-0 and *OE2*, *OE3* were evaluated via qRT-PCR. *AtACTIN 2* was used as the internal control. Primers used are listed in Table S1. For **(B)**, asterisks indicate significant differences from Col-0. Data shown are means of three independent biological replicates (*P<0.05, one way ANOVA).

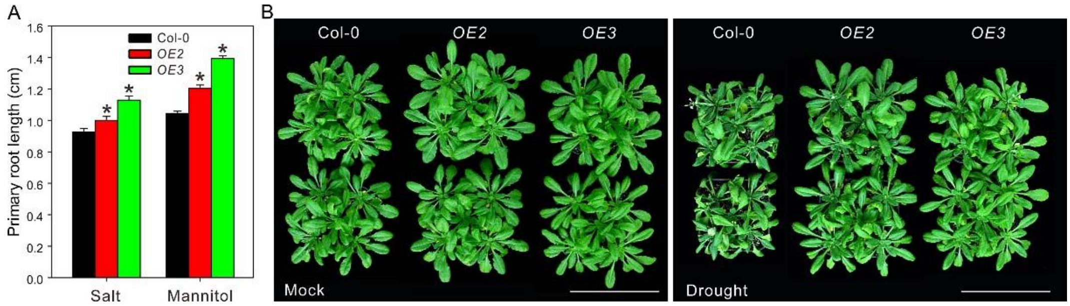

Figure S3. *ZxPIP1;3* overexpression improves salt and drought tolerance. **(A)** Primary root length of wild type (Col-0) and *ZxPIP1;3* overexpression lines (*OE2* and *OE3*) on 1/2 MS solid medium with 150 mM NaCl (Salt) or 300 mM mannitol (Mannitol). **(B)** Phenotypes of 5-week-old Col-0 and *OE2*, *OE3* under well-watered condition (Mock) and 7-day drought treatment (Drought). Bar = 10 cm. For **(A)**, asterisks indicate significant differences from Col-0 (n = 13 per column. *P < 0.05, one way ANOVA).

Table S1. Primers used in current study.

Primers	Sequences
ZxPIP1;3-3'Fw	CACAAGGTATGAGCTTCTTG
ZxPIP1;3-3'Fn	ACTGTGTTCGCTGCCACTG
ZxPIP1;3-5'Rw	GTTGAAGATGATGGCAGCTC
ZxPIP1;3-5'Rn	CAGCGCCAATGAATGGTCCGA
ZxPIP1;3-FULL-F	AAAAAGCAGGCTtcATGGAAGGAAAAGAAGAGGATGTG
ZxPIP1;3-FULL-R	AGAAAGCTGGGTtTGCCCTGGTCTTGAATGGAATG
ZxPIP1;3-QF	CCAGCAGTGACATTTGGGTTG
ZxPIP1;3-QR	GCACCAAGACACTGCATCAC
ZxPIP1;3-FLAG-semiQF	CACTAAGGGTGATGGTCTTGGTG
ZxPIP1;3-FLAG-semiQR	CTTATCGTCGTCATCCTTGT
ZxPIP1;3-FLAG-QF	ATGACCACTGGATCTTCTGG
ZxPIP1;3-FLAG-QR	CTTATCGTCGTCATCCTTGT
AtP5CS1-QF	ATCCCTGTGCTAGGTCATGC
AtP5CS1-QR	CTATGCGCTTTGCCATATCCG
AtRD29A-QF	GATGGAAGATTCTGTCTCAACGAT
AtRD29A-QR	GTTTCTCCTTCACTATCTCCTCCG
AtDREB1A-QF	GAGATGTGTGATGCGACGAC
AtDREB1A-QR	CTCAAACATCGCCTCATCGTG
ZxACTIN-QF	TTTTCCAGCCATCCCTTGTT
ZxACTIN-QR	TGCAGTGATCTCCTTGCTCATAC
AtACTIN 2-QF	TCAGATGCCCAGAAGTGTTGTTCC
AtACTIN 2-QR	CCGTACAGATCCTTCCTGATATCC
AtACTIN 2-semiQF	TCAGATGCCCAGAAGTCTTGTT
AtACTIN 2-semiQR	GAGATCCACATCTGCTGGAATG
